# Taking the STING out of radiotherapy: STING checkpoints mediate radiation resistance

**DOI:** 10.1172/JCI186547

**Published:** 2024-12-02

**Authors:** Michael C. Brown, Justin T. Low, Michelle L. Bowie, David M. Ashley

**Affiliations:** The Preston Robert Tisch Brain Tumor Center, Department of Neurosurgery, Duke University School of Medicine, Durham, North Carolina, USA.

## Abstract

The cyclic GMP-AMP synthase/stimulator of interferon genes (cGAS/STING) pathway is a critical driver of type I interferon (IFN-I) and antitumor CD8^+^ T cell responses after radiotherapy (RT). In this issue of the *JCI*, two reports describe mechanisms that restrained STING signaling and abrogated antitumor immunity after RT. Wen, Wang, and colleagues discovered that IFN-I mediated the induction of YTHDF1, an RNA *N^6^*-methyladenosine–binding protein, in DCs after RT promoted cathepsin-mediated STING degradation. Zhang, Deng, Wu, and colleagues discovered that hemeoxygenase 1 (HO-1) was induced and proteolytically cleaved after RT to suppress cGAS cytoplasmic export as well as STING oligomerization at the ER. Blocking the STING-suppressive functions of YTHDF1 and HO-1, respectively, improved antitumor T cell immunity and tumor control after RT. Together, these studies support the development of clinical avenues to sustain STING signaling during RT, a standard treatment for approximately 50% of malignancies.

## Radiotherapy and STING signaling in antitumor T cell immunity

Pattern recognition receptors (PRRs) such as cyclic GMP-AMP synthase (cGAS) and stimulator of interferon genes (STING) dictate adaptive immune responses by coordinating antigen presentation, immune cell recruitment, T cell costimulation, and T cell priming. Activation of the cGAS/STING pathway stokes antitumor immunity by sensing cytosolic double-stranded DNA (dsDNA) from engulfed cancer cells, dsDNA released from nuclear and/or mitochondrial damage, and/or damaged dsDNA in cytosolic micronuclei (1). Cell-to-cell transfer of cyclic dinucleotides (e.g., 2′3′-cyclic GMP-AMP [cGAMP]), which are produced by cGAS upon dsDNA sensing, also enables STING activation in a paracrine manner (1). In mice, STING signaling is required for the generation of spontaneous antitumor immunity (2) and mediates antitumor immune responses in an type I interferon–dependent (IFN-I–dependent) manner after radiotherapy (RT) (3). Indeed, IFN-I signaling in DCs is required for antitumor T cell priming (4) and the antitumor efficacy of RT in mice (5). Thus, it is not surprising that genetic and epigenetic suppression of cGAS and STING has been observed in several cancer types (6, 7). Moreover, during infectious and inflammatory processes, PRR-induced inflammation must be resolved or tempered to prevent excessive tissue damage and enable homeostasis. Numerous examples of such mechanisms have been demonstrated (e.g., dephosphorylation of IRF3 [ref. 8] or degradation of STING [ref. 9]). Yet, whether blocking negative feedback mechanisms, which presumably evolved to prevent overzealous innate immune responses, can mediate cancer control has not widely been explored. Analogous to targeting immune checkpoints (e.g., programmed death ligand 1 [PD-L1]) that temper adaptive immune responses, blocking negative regulators of STING signaling may hold therapeutic promise by sustaining the cues that allow for CD8^+^ T cell priming and function, particularly after RT. Since approximately half of all malignancies are treated with RT, and RT mediates in situ cancer vaccination (i.e., priming of antitumor T cells) by inducing STING signaling, identifying routes to maximize STING-induced IFN responses to RT may yield tremendous clinical value.

## YTHDF1 and HO-1 suppress STING signaling after RT

In this issue of the *JCI*, two reports (10, 11) define how distinct mechanisms, induced by RT, restrain STING signaling and can be blocked to amplify the immune-engaging and antitumor effects of RT.

The RNA *N^6^*-methyladenosine binding protein YTHDF1 suppresses tumor antigen cross-presentation on DCs by stabilizing lysosomal cathepsin encoding mRNAs (12). Wen, Wang, and colleagues (10) discovered that YTHDF1 was induced in peripheral blood DCs and associated with shorter progression-free survival of patients with non–small cell lung cancer after RT. The induction of YTHDF1 was dependent on IFN-I signaling and mediated by STAT2, downstream of the IFN α/β receptor (IFNAR). *Ythdf1* deletion enhanced IFN-I production in DCs, and DC-specific deletion of *Ifnar* negated the antitumor effects of *Ythdf1* deletion after RT. It was previously demonstrated that YTHDF1 stabilizes lysosomal cathepsins (12) and that STING signaling occurs in vesicles that are degraded by lysosomes after activation (13, 14). Wen et al. reported several effects of RT: cathepsins were induced along with YTHDF1, STING physically interacted with cathepsins, and STING signaling and associated IFN responses increased after cathepsin inhibition in vitro and in vivo (10). Importantly, two strategies improved tumor control: in vivo cathepsin inhibition combined with RT, as well as *Ythdf1* deletion or inhibition in tumor antigen–loaded DC vaccines delivered to mice receiving RT (10). Thus, YTHDF1 mediates immunological resistance to radiation, and targeting YTHDF1 or cathepsins to prolong STING signaling after RT may represent a route to improve patient outcomes.

Zhang, Deng, Wu and colleagues (11) sought to discover genes that determine IFN induction after RT using a CRISPR-KO screen targeting metabolic genes in a nasopharyngeal cancer cell line. Deletion of the antioxidant hemeoxygenase 1 (HO-1) potently promoted IFN-β production after RT. Inducible knockdown of HO-1 in various murine models sensitized tumors to RT. Using RNA silencing and analysis of cell signaling via immunoblotting, the authors determined that HO-1 ablation promoted IFN-I via cGAS/STING signaling. Intriguingly, HO-1 ablation increased cGAMP production after RT, but also enhanced IFN-β production after cGAMP treatment, implying that HO-1 acts on cGAS/STING signaling at multiple nodes. Expression of HO-1 has previously been shown to be promoted by ROS, and HO-1 is believed to mediate antiinflammatory effects primarily through the action of its enzymatic products (15). However, RT and IFN-β induced HO-1 expression independent of ROS, and HO-1 suppressed IFN-I induction after RT independent of its catalytic activity. Induction of full-length and cleaved versions of HO-1 were observed after RT; with cleavage of HO-1 only being observed after RT (11). Using truncated and mutant HO-1 variants, the authors discovered that full-length HO-1 remained anchored to the ER via its transmembrane domain, while RT-induced HO-1 cleavage disrupted the transmembrane domain of HO-1, causing its nuclear relocalization. Nuclear (truncated) HO-1 directly bound to cGAS to prevent its nuclear export after RT, thereby limiting the production of STING-activating cGAMP in the cytosol. Independent of this function, full-length (uncleaved) HO-1 was retained at the ER, where it was shown to directly interact with STING, prevent its oligomerization, and preclude its association with TBK1, the kinase responsible for IRF3 phosphorylation and subsequent IFN production after STING activation. A screen for inhibitors of HO-1 that potentiated RT-induced IFN-β production yielded a candidate (HO-1–IN-1) that promoted cGAMP production and STING activation. This inhibitor promoted antitumor and immunogenic effects of RT in vivo, demonstrating preclinical feasibility of targeting HO-1 to augment STING-mediated inflammation after RT. HO-1 expression was associated with poor outcomes for patients with nasopharyngeal cancer and shorter survival in patients with brain cancer treated with RT. This work identifies HO-1 as a targetable mechanism that restrains STING signaling after RT and, more broadly, implicates HO-1 as a regulator of STING signaling.

**Figure 1:**
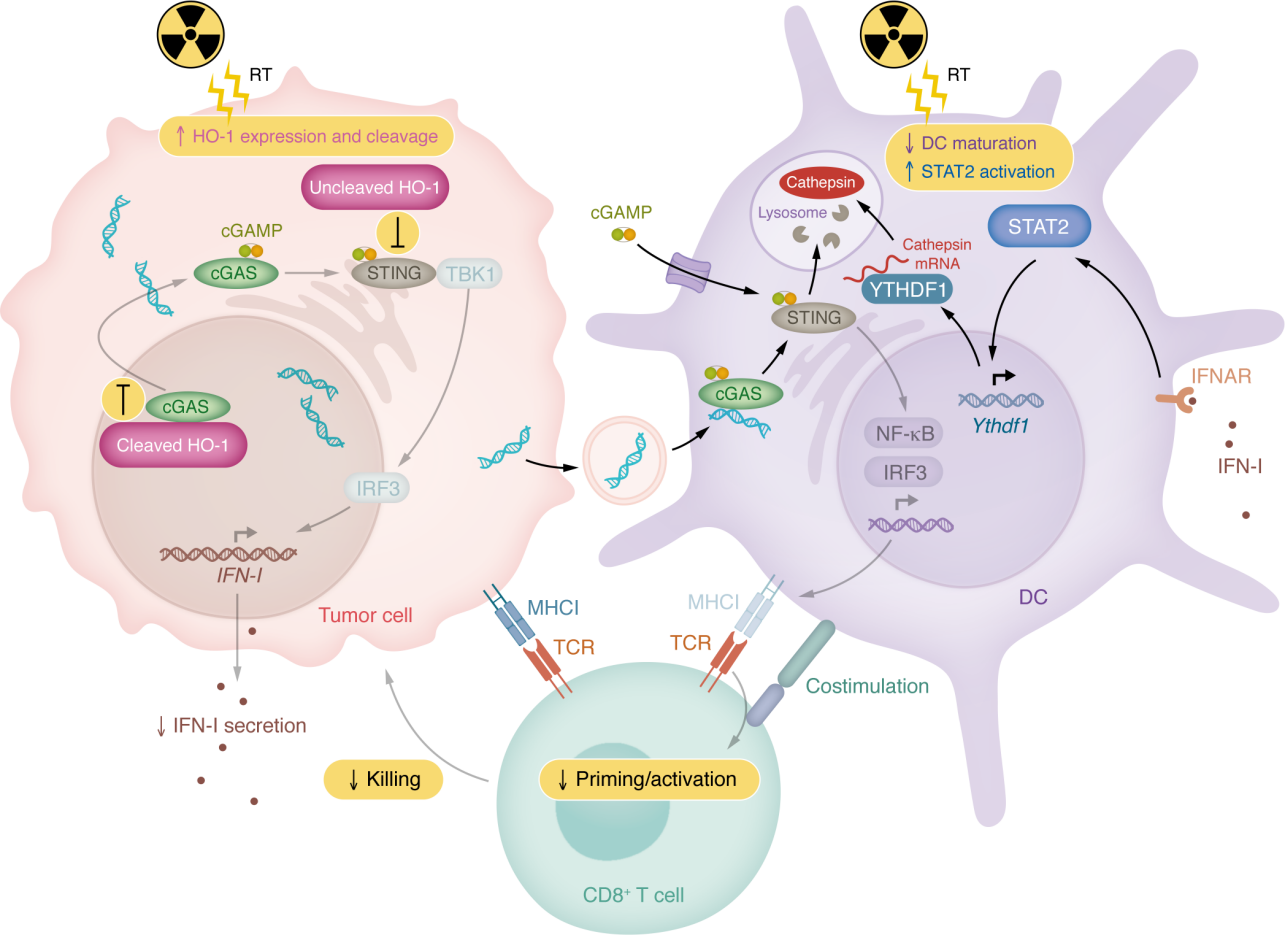
STING suppression after RT limits antitumor T cell immunity. Zhang et al. (11) demonstrated that RT induces HO-1 expression and cleavage in cancer cells. This process leads to two mechanisms by which HO-1 suppresses cGAS/STING signaling. First, cleaved HO-1 localizes to the nucleus and binds to cGAS to prevent its nuclear export — preventing cGAS-dependent production of cytosolic cyclic dinucleotides (CDNs), such as cGAMP, that activate STING. Second, uncleaved HO-1, which retains its transmembrane domain, remains at the ER and directly interacts with STING to prevent its oligomerization, ER lumen curvature, and interaction with TBK1, impeding downstream signaling from STING. Together, these effects reduce the amount of intracellular CDN production and IFN-I secretion, limiting the delivery of these key immunostimulatory molecules to DCs and other cells within the tumor microenvironment. Wen et al. (10) discovered that RT induces the expression of YTHDF1 through IFN-I:IFNAR–dependent STAT2 activation, which directly binds to the *Ythdf1* promoter to promote YTHDF1 transcription in DCs. YTHDF1 then binds to cathepsin mRNA to support its translation, leading to an overall increase in cathepsin expression and presence in lysosomes. STING activation, elicited by either engulfed cancer cell DNA recognized by cGAS or through import of extracellular CDNs, leads to the oligomerization of STING and production of vesicles from which productive STING signaling occurs. These vesicles are degraded by cathepsins in lysosomes, attenuating STING signaling. Ultimately, cancer cell and DC-intrinsic STING signaling can induce DC maturation/activation that leads to priming of tumor antigen–specific CD8^+^ T cells by DCs that recognize and kill cancer cells. Both HO-1 and YTHDF1 impair DC maturation, cross-presentation, and/or antitumor T cell immunity after radiation.

Both studies (10, 11) identify compelling mechanisms, supported by murine models and associated human correlatives, that impede RT-induced STING signaling and, thus, antitumor T cell immunity. The authors of each study demonstrated proof of principle that STING signaling can be augmented to boost in situ vaccination effects of RT: either by blocking cathepsin-mediated degradation of active STING signaling complexes in DCs (10), or by blocking the suppressive function of HO-1 on both cGAS and STING in cancer cells (11) (Figure 1).

## Future considerations and conclusions

Together, these studies underscore the role of cGAS/STING signaling in mediating the antitumor benefit of RT and strongly imply that such benefit could be improved with protracted STING signaling. Yet, paradoxically, STING and IFN signaling has also been shown to mediate radiation resistance, immunotherapy resistance, and T cell exhaustion/dysfunction (16–19). Such apparent discrepancies may reflect context-dependent effects of STING signaling on antitumor immunity. For example, the broader inflammatory context associated with STING-induced IFN responses may influence whether productive antitumor immunity is engaged. Other factors may also modulate STING-driven immune responses in the tumor microenvironment, including the cellular compartment(s) in which STING and/or IFN signaling are induced (e.g., DCs or malignant cells), the nature of antitumor T cell specificities and functionalities, and the density of antigen-presenting cells within the tumor. Wen, Wang, and colleagues pin-pointed the effect of YTHDF1-mediated STING suppression to DCs (10). While it has been shown that STING signaling in DCs is sufficient to mediate antitumor immunity (1), it remains unclear if STING engagement in other contexts (e.g., in malignant cells) can also promote immune surveillance (e.g., via secreted IFN that signals through IFNAR on DCs). cGAS and STING have been shown to be silenced in several cancer types (6, 7), suggesting a potential antitumor role for tumor-intrinsic cGAS/STING signaling. While Zhang, Deng, Wu and colleagues demonstrated that tumor-specific ablation of HO-1 could potentiate RT in vivo through STING (11), the observed antitumor effects in vivo could have been mediated by transfer of extracellular cGAMP produced by cancer cells to trigger STING in DCs within the tumor microenvironment (1). Thus, whether STING signaling in compartments other than DCs engages immune surveillance, as well as the contexts in which STING and IFN signaling promotes antitumor immunity as opposed to contributing to therapy resistance, remain critical unanswered questions.

Beyond radiation, and despite a lack of momentum from early-stage clinical trials (1), targeting the intratumor cGAS/STING axis with specific agonists of STING (e.g., analogs or derivatives of cGAMP) remains of interest in immunologically quiescent cancer types, such as gliomas (20). Radiation has the advantages of being delivered iteratively without invasive intratumor injections, mediating antitumor activity through direct tumor cell killing and causing STING activation in the context of malignant cell death — which may be more immunogenic than STING signaling in isolation. However, RT also leads to death of possibly beneficial tumor-infiltrating immune cells, and intratumor STING agonist therapy may achieve stronger STING signaling than that induced by RT. In sum, RT is likely distinct in its immune-engaging qualities from those of STING agonists and is also more clinically feasible to incorporate into combination regimens, given the existing widespread use of RT for cancer. Therefore, prolonging STING signaling during RT using approaches such as those described in this issue (10, 11), is a compelling strategy to leverage the in situ vaccination potential of cGAS/STING signaling clinically.

## Version 1 12/02/2024

Electronic publication
